# Extracorporeal Photopheresis With Low-Dose Immunosuppression in High-Risk Heart Transplant Patients—A Pilot Study

**DOI:** 10.3389/ti.2022.10320

**Published:** 2022-03-23

**Authors:** Johannes Gökler, Arezu Aliabadi-Zuckermann, Andreas Zuckermann, Emilio Osorio, Robert Knobler, Roxana Moayedifar, Philipp Angleitner, Gerda Leitner, Günther Laufer, Nina Worel

**Affiliations:** ^1^ Department of Cardiac Surgery, Medical University of Vienna, Vienna, Austria; ^2^ Department of Dermatology, Medical University of Vienna, Vienna, Austria; ^3^ Department of Blood Group Serology and Transfusion Medicine, Medical University of Vienna, Vienna, Austria

**Keywords:** extracorporeal photopheresis, heart transplantation, immunosuppression, CNI delay, induction therapy

## Abstract

In severely ill patients undergoing urgent heart transplant (HTX), immunosuppression carries high risks of infection, malignancy, and death. Low-dose immunosuppressive protocols have higher rejection rates. We combined extracorporeal photopheresis (ECP), an established therapy for acute rejection, with reduced-intensity immunosuppression. Twenty-eight high-risk patients (13 with high risk of infection due to infection at the time of transplant, 7 bridging to transplant via extracorporeal membrane oxygenation, 8 with high risk of malignancy) were treated, without induction therapy. Prophylactic ECP for 6 months (24 procedures) was initiated immediately postoperatively. Immunosuppression consisted of low-dose tacrolimus (8–10 ng/ml, months 1–6; 5–8 ng/ml, >6 months) with delayed start; mycophenolate mofetil (MMF); and low maintenance steroid with delayed start (POD 7) and tapering in the first year. One-year survival was 88.5%. Three patients died from infection (POD 12, 51, 351), and one from recurrence of cancer (POD 400). Incidence of severe infection was 17.9% (*n* = 5, respiratory tract). Within the first year, antibody-mediated rejection was detected in one patient (3.6%) and acute cellular rejection in four (14.3%). ECP with reduced-intensity immunosuppression is safe and effective in avoiding allograft rejection in HTX recipients with risk of severe infection or cancer recurrence.

## Introduction

Cardiac transplantation remains the best option for patients with end-stage heart failure. In recent decades, the number of patients referred to transplantation has increased significantly. Many patients are at high risk for early postoperative infection, and patients with previous malignant disease are more often seen as potential transplant candidates (1, 2). Current immunosuppressive protocols are associated with risk of infectious complications and cancer (3, 4). Earlier attempts to use low-level immunosuppressive protocols to reduce these risks resulted in higher organ rejection rates (5, 6). Extracorporeal photopheresis (ECP) is a successful supportive therapy for the treatment of severe and/or recurrent rejection episodes in solid organ transplantation, including heart transplantation (7). ECP is an apheresis involving ultraviolet A irradiation of peripheral blood mononuclear cells with prior exposure to 8-methoxypsoralen. The mode of action is not fully understood, but ECP is believed to have immunostimulatory and immunosuppressive effects and it reduces T-cell-mediated immune responses (8). In 1998, Barr *et al.* published a landmark prospective randomized study that documented the benefit of ECP as adjunct to standard immunosuppression to prevent acute rejection (14). However, the applied immunosuppressive protocol (cyclosporine A, azathioprine) differs from the protocols used today.

The aim of our pilot study was to evaluate a novel approach consisting of 6-month ECP together with a reduced-intensity immunosuppressive protocol to treat challenging heart transplant recipients at high risk for either early postoperative infection or cancer recurrence.

## Materials and Methods

### Study Design

The primary outcomes of this pilot study were 1-year and overall survival. Secondary outcomes were the safety of ECP, incidence of early postoperative infection (in-hospital and in the first 6 months of ECP therapy), number of rejection episodes according to the International Society of Heart and Lung Transplantation (ISHLT) criteria in the first year, and recurrence of malignancy. Approval for the study was obtained from the institutional review board of the Medical University of Vienna (EK 1107/2020). In accordance with local regulations, all use of patients’ clinical research data required their consent.

### Patients

Between September 2016 and January 2021, 200 heart transplant procedures were performed at the Medical University of Vienna. Twenty-eight patients (25% female, *n* = 7) were included in this study and treated according to our reduced-intensity immunosuppressive protocol without induction but combined with ECP. There was no adequate control group to compare with this heterogeneous pilot group of challenging HTX patients. Most patients (85.7%) had highly urgent status. Inclusion criteria for this protocol consisted of patients with a recent or current history of infection (patients with sepsis or systemic inflammatory response syndrome were excluded, as these are absolute contraindications for transplantation in our center), high risk for early postoperative infection (ECMO bridging to transplant), or high neoplastic risk (i.e., cardiac tumor as indication for transplantation, history of malignancy more than 5 years prior to transplantation, malignancy found in the donor after organ procurement).

Patient demographics and baseline characteristics are listed in Tables 1, 2. Detailed information on indication for inclusion in the study protocol is presented in Table 3.

**TABLE 1:** Patient demographics and baseline characteristics I.

	Total, *n* = 28	Infection, *n* = 13	ECMO, *n* = 7	Malignancy, *n* = 8
Age, years, med (IQR)	51.9 (42.2–57.6)	55.7 (52.5–63.4)	43 (37.2–51.8)	43.8 (39.5–51.4)
Gender, female, n (%)	7 (25)	2 (15.4)	1 (14.3)	4 (50)
Indication for HTX, n				
Ischemic CMP	5	3	2	0
Dilative CMP	10	5	0	5
Congenital disease	1	1	0	0
Bail out after cardiac surgery	6	1	5	0
Cardiac tumor	2	0	0	2
Other (CAV, HOCM)	4	3	0	1
HKTX, n (%)	2 (7.1)	1 (7.7)	0	1 (12.5)
Previous cardiac surgery, n (%)	18 (64.3)	7 (53.8)	5 (71.4)	6 (75)

CAV, cardiac allograft vasculopathy; CMP, cardiomyopathy; HKTX, combined heart-kidney transplant; HTX, heart transplantation; HOCM, hypertrophic obstructive cardiomyopathy; med (IQR), median and interquartile range.

**TABLE 2:** Patient demographics and baseline characteristics II.

	Total, *n* = 28	Infection, *n* = 13	ECMO, *n* = 7	Malignancy, *n* = 8
High urgency status, n (%)	24 (85.7)	12 (92.3)	7 (100)	5 (62.5)
IMPACT score, med (IQR)	8 (5.8–13)	7 (6–10)	14 (12.5–16.5)	4.0 (2.5–7.8)
ICU, n (%)	14 (50)	6 (46.2)	7 (100)	1 (12.5)
Intubated, n (%)	3 (10.7)	0	3 (42.9)	0
Infection, n (%)	20 (71.4)	13 (100)	7 (100)	0
ECMO support, n (%)	7 (25)	0	7 (100)	0
VAD, n (%)	7 (25)	5 (38.5)	0	2 (25)
eGFR, med (IQR)	84.7 (36.9–104.2)	93.4 (35–100.9)	120 (69.8–174.1)	67.3 (29.6–84.7)
Creatinine, mg/dl, med (IQR)	1.1 (0.8–1.8)	1.2 (0.9–1.9)	0.6 (0.5–1)	1.2 (1–2.2)
RRT, n (%)	6 (21.4)	3 (30)	2 (28.6)	1 (12.5)
Bilirubin, (mg/dl), med (IQR)	0.8 (0.5–1.1)	0.8 (0.5–1.2)	1 (0.8–2)	0.5 (0.4–0.8)
Diabetes (IDDM), n (%)	4 (14.3)	3 (23.1)	0	1 (12.5)

ECMO, extracorporeal membrane oxygenation; eGFR, estimated glomerular filtration rate; ICU, intensive care unit; IDDM, insulin-dependent diabetes mellitus; IMPACT, index for mortality prediction after cardiac transplantation; med (IQR), median and interquartile range; RRT, renal replacement therapy; VAD, ventricular assist device.

**TABLE 3:** Indication for ECP.

Infection *n* = 13 (46%)	Microbiological result	Site of infection at time of HTX	
1	*Staph. haemolyticus*/*epidermidis*	Blood culture, postop sternal VAC and ECMO	
2	*E. faecalis*	Site of kidney transplant with postop local VAC therapy	
3	*Staph. epidermidis*	Blood culture	
4	*Klebsiella pn*., *Proteus mirabilis*	Ascites	
5	*Staph. aureus*	Blood culture	
6	Hepatitis B PCR +	Blood culture; HTX in deep hypothermia with circulatory arrest	
7	*E. coli*	Recurrent endocarditis, BAL	
8	*Staph. aureus*	Blood culture	
9	*P. aeruginosa* 4MRGN	Blood culture, driveline, mediastinum	
10	*Staph. aureus*	Blood culture, mediastinum	
11	*P. aeruginosa*	Blood culture, driveline, mediastinum	
12	*Citrobacter koseri* ESBL, *Aspergillus fumigatus*	Fungal sinusitis	
13	*Staph. lugdunensis*	Blood culture, driveline	
**ECMO *n* = 7 (25%)**	**Cause of ECMO**	**Detail**	**Days on ECMO before HTX**
1	Post cardiotomy	Mech Bentall procedure; LVAD; LVAD explant	5
2	Myocardial infarction	STEMI with PCI, ischemic ventricular rupture	14
3	Post cardiotomy	MV-repair and AVR	25
4	Post cardiotomy	STEMI, CABG	27
5	Post cardiotomy (endocarditis)	Mitral and aortic valve replacement, CABG (CX)	17
6	Post cardiotomy	Type A dissection (mech Bentall)	23
7	Right heart failure	CMP with decompensation	1
**Malignancy *n* = 8 (29%)**	**Histology**	**Interval between diagnosis and HTX**	**Complete remission**
1	Myxofibrosarcoma heart	12 months	no
2	Synovial sarcoma heart	6 months	no
3	Osteosarcoma; breast cancer (recurrence)	30 years; 12 years (8 years)	yes
4	PTLD (HTX)	10 years	yes
5	Renal cell carcinoma	10 years	yes
6	ALL; cerebral recurrence	13 years; 5 years	yes
7	Non-Hodgkin’s lymphoma	42 years	yes
8	Adenocarcinoma in donor lung	0	yes

ALL, acute lymphoblastic leukemia; AVR, aortic valve replacement; BAL, bronchoalveolar lavage; CABG, coronary artery bypass graft; CX, circumflex artery; CMP, cardiomyopathy; ECMO, extracorporeal membrane oxygenation; ECP, extracorporeal photopheresis; *E. coli*, *Escherichia coli*; *E. faecalis*, *Enterococcus faecalis*; HTX, heart transplantation; IQR, interquartile range; LVAD, left ventricular assist device; *Klebsiella pn*., *klebsiella pneumoniae*; mech, mechanical; MV, mitral valve; PCI, percutaneous coronary intervention; PCR, polymerase chain reaction; *P. aeruginosa*, *Pseudomonas aeruginosa*; postop, postoperative; PTLD, post-transplant lymphoproliferative disorder; *Staph.*, *Staphylococcus* STEMI, ST-elevation myocardial infarction; VAC, vacuum assisted closure.

### Outcome Parameters

Postoperative severe infection was defined as clinically relevant infection in the early postoperative phase. CMV disease was based on international classification (9).

Graft function was examined by transthoracic echocardiography, which was performed on a routine basis during the first year (weekly in month 1, monthly in months 2–12). Endomyocardial biopsies were performed at weeks 2, 3, and 4, and at months 2, 3, 6, and 12, and in case of clinical signs of rejection. Acute cellular rejection (ACR) as well as antibody-mediated rejection were defined according to the ISHLT nomenclature (10, 11).

Patients with a history of malignancy underwent close follow-up including CT, MRI, or PET scan where appropriate, on a regular basis.

### Adjusted Immunosuppressive Protocol

**FIGURE 1:**
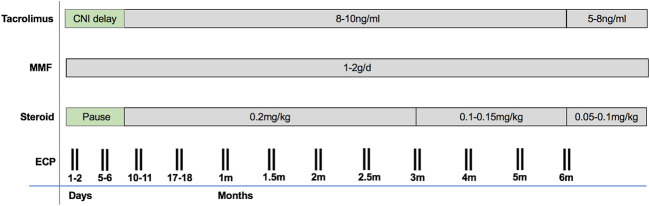
An overview on our immunosuppressive protocol including ECP.

There was no induction therapy (see Figure 1). For immunosuppression, the calcineurin inhibitor (CNI) tacrolimus was first administered after a CNI delay of at least 3 days in patients with normal renal function and up to 10 days in patients with reduced kidney function. The target range of tacrolimus was 8–10 ng/ml in months 1–6, and 5–8 ng/ml thereafter. Mycophenolate mofetil was started on postoperative day 0 with 1 g/day and increased to 2 g/day at the time of CNI start, in case of normal leukocyte counts (>4000 per microliter). After postoperative wound healing, MMF was switched to everolimus (starting dose 1.5 mg/d; through level 8 ng/ml) in the patients of the malignancy group due to its potential antineoplastic effects (12). Steroid was applied intraoperatively (500 mg methylprednisolone prior to opening the aortic clamp) and in the first 24 h (125 mg methylprednisolone every 8 h). Maintenance steroid (0.2 mg/kg/day prednisolone) was started on POD 7 and tapered by 2.5 mg every 3 months in the absence of rejection.

### Prophylaxis of Infection

All patients without evidence of infection at the time of HTX received empiric broad-spectrum antibiotics for at least 5 days after transplantation, and all patients with infection at the time of transplantation were treated with targeted antimicrobial therapy adjusted to the antibiogram. Prophylaxis against *Pneumocystis jerovecii* with oral trimethoprim-sulfamethoxazole (160 mg trimethoprim and 800 mg of sulfamethoxazole, two tablets per day, 3 times per week) was given for 6 months. CMV prophylaxis consisted of 100 ml of anti-CMV hyper-immunoglobulin (Cytotect; Biotest Pharmaceuticals Corporation, Boca Raton, Florida) on POD 1, 7, 14, and 28, and patients at high risk for CMV infection (recipient CMV antibody negative and donor CMV antibody positive) received oral valganciclovir (900 mg/day) for 3 months. CMV infection was monitored using PCR for CMV DNA, and patients with CMV DNA >1000 copies/mL on any PCR test were treated pre-emptively with valganciclovir adjusted according to their renal function.

### ECP Protocol

ECP therapy was based on the previously published protocol by Barr et al. (13), consisting of a total of 24 ECP procedures during a 6-month period starting immediately after transplantation as follows: on POD 1 and 2, 5 and 6, 10 and 11, 17 and 18, 27 and 28, on two consecutive days every other week in months 2 and 3, and on two consecutive days once a month in months 4–6 (13). ECP was performed using the Cellex Photopheresis System (Therakos Ltd.; Mallinckrodt Pharmaceuticals) with either double- or single-needle access. Briefly, during an ECP session, 1500 ml of whole blood was processed, and peripheral blood mononuclear cells (MNCs) were separated by centrifugation (14). After MNC collection, the photosensitizer 8-methoxypsoralen (Uvadex) at a dose of 20 μg/ml was added to the MNC collection bag and cells were irradiated with ultraviolet A light (1.5 J/m^2^) before being returned to the patient. For anticoagulation, acid citrate dextrose A was used at a ratio of 1:10 to avoid bleeding complications.

### Statistical Analyses

Data including demographic and transplant variables were obtained from the Medical University of Vienna Heart Transplant Database. The statistical analyses were performed using the Statistical Program of Social Sciences 22.0 (SPSS Inc., Chicago, IL United States). Categorical variables are described by absolute and relative frequencies, and continuous variables by median and interquartile range (IQR). The Kaplan-Meier estimate was used for survival analysis. P-values below 0.05 were considered statistically significant.

## Results

### Survival

One-year survival in these high-risk recipients was 88.5% by Kaplan-Meier estimate (25/28 patients). Infectious complications leading to septic multiorgan failure (MOF) were the cause of death in three patients on POD 12, 51, and 351, respectively. One patient with a malignant tumor of the heart as transplant indication died due to recurrence of malignancy 400 days after HTX. Therefore, overall survival in our cohort was 84.0% (*n* = 24) with a median follow-up of 23.7 months (IQR 12.7–33.4). Considering the different indications for ECP, patients with pre-transplant infection had the highest mortality rate of 23% (3/13), patients with malignancy 12.5% (1/8), and there were no deaths in patients bridged to HTX with ECMO (see Table 4). The non-ECP cohort transplanted during the study period (*n* = 172) had an estimated 1-year survival rate of 93%.

**TABLE 4:** Outcome variables.

	Total, *n* = 28	Infection, *n* = 13	ECMO, *n* = 7	Malignancy, *n* = 8
1-year survival, n (%)	25 (88.5)	10 (75)	7 (100)	8 (100)
Overall survival, n (%)	24 (84.0)	10 (75)	7 (100)	7 (87.5)
Follow-up, m, med (IQR)	23.7 (12.7–33.4)	23.6 (8.4–32.3)	30.7 (18.9–38.8)	24.1 (13.8–43.0)
ICU stay, d, med (IQR)	17.5 (10.8–31.8)	17.5 (10.5–29.5)	30 (15–32.5)	17.5 (10.5–29.5)
In hospital stay, d, med (IQR)	43 (32–55)	39.5 (32.5–54.3)	43.5 (35.5–54.5)	39.5 (32.5–54.3)
RRT, n (%)	13 (46.4)	8 (61.5)	2 (28.6)	3 (37.5)
Pneumonia, n (%)	5 (17.9)	3 (23.1)	0	2 (25)
Sepsis, n (%)	2 (7.1)	2 (15.4)	0	0
ACR≥2R in the first year, n (%)	4 (14.3)	1 (7.7)	1 (14.3)	2 (25)
AMR	1 (3.6)	1 (7.7)	0	0
PGD grade 3, n (%)	2 (7.1)	2 (15.4)	0	0

ACR, acute cellular rejection; AMR, antibody-mediated rejection; d, days; ICU, intensive care unit; med (IQR), median and interquartile range; m, months; PGD, primary graft dysfunction with grading according to the ISHLT, consensus 2014; RRT, renal replacement therapy.

### Immunosuppressive Protocol

**FIGURE 2:**
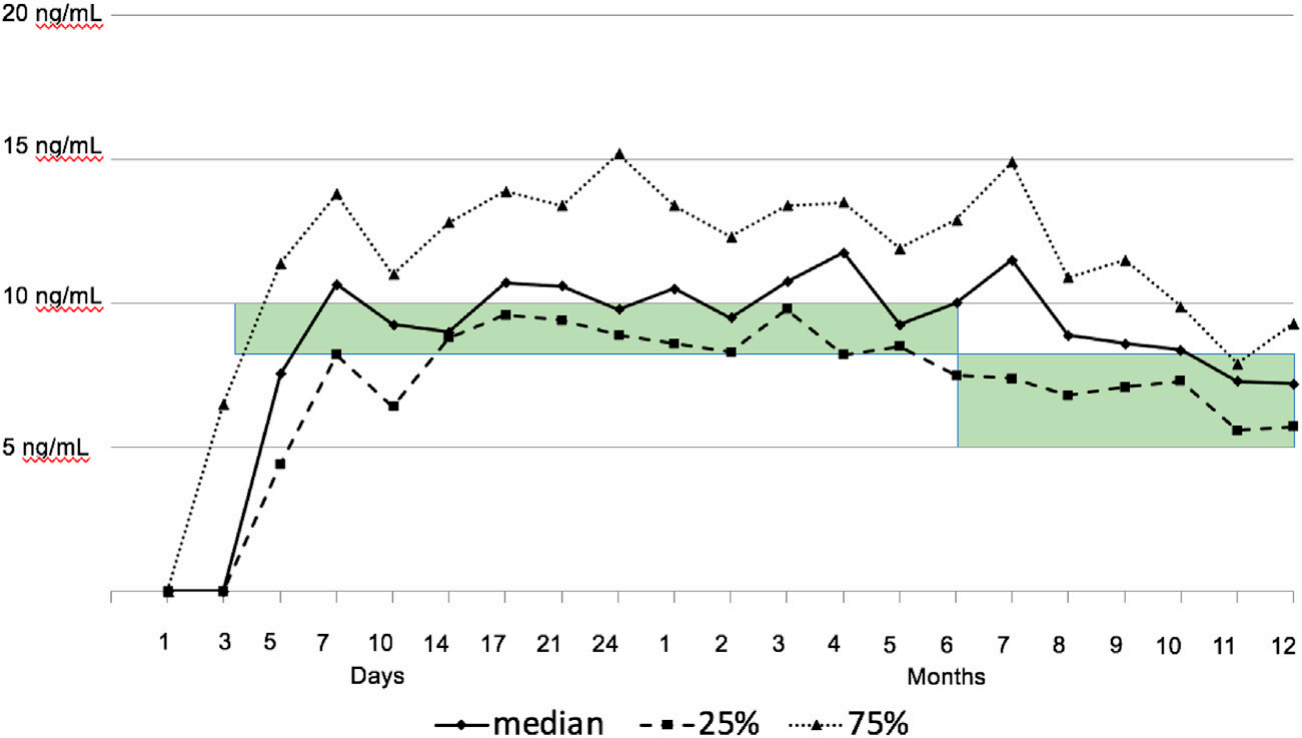
Tacrolimus trough levels. The green bar highlights the intended target range of tacrolimus (8–10 ng/ml in months 1–6, and 5–8 ng/ml thereafter).

CNI delay was achieved in all patients with a median start time of tacrolimus on POD 3 (IQR 2–4), and the longest CNI delay was 9 days in one patient. Target tacrolimus trough levels were attained for the whole patient cohort (see Figure 2). MMF was started on POD 0 in all patients. MMF was switched to everolimus in five patients of the malignancy group (62.5%). Steroids were given as described above. In our first two patients, a single dose of induction therapy with 100 mg of rabbit anti-thymocyte globulin (ATG) was given on POD 1.

All patients who survived the first 6 months received ECP according to the protocol. Overall, ECP was tolerated well. In one patient, elevated potassium levels occurred during the third ECP treatment and could not be attributed to ECP. Most likely, intravenous amphotericin A was administered too quickly, causing a shift of potassium (15, 16). Due to invasive fungal infection, maintenance immunosuppression (CNI, MMF, and steroid) and ECP were paused in two patients who died of sepsis leading to MOF on POD 12 and 51, respectively (see below).

### Postoperative Infections

Severe bacterial (*n* = 3) and fungal (*n* = 2) infections emerged in five patients (17.9%) in the immediate postoperative period (see Table 4). All were lower respiratory tract infections necessitating either prolonged ventilation (*n* = 3) or reintubation (*n* = 2). The three patients with bacterial infections were successfully weaned from ventilation after targeted antimicrobial therapy. The two with invasive fungal infections died due to sepsis and MOF on POD 12 and 51. The identified pathogens in bronchoalveolar lavage and blood cultures were *Aspergillus niger* and *Candida albicans*, respectively. In both cases, the fungal strains were resistant to empirically administered antifung4al therapy. CMV disease with enteritis occurred in one CMV high-risk (D+/R-) patient 2 months after HTX, after prophylaxis with valganciclovir had been discontinued. However, the patient was successfully treated with valganciclovir in therapeutic dosage for 2 weeks. No other CMV infection was detected.

### Sensitization and Rejection

Three patients showed sensitization prior to transplantation, with calculated panel-reactive antibodies of 23%, 51% and 67%, and were transplanted *via* negative virtual crossmatch, which was confirmed by negative complement-dependent cytotoxicity (CDC) crossmatch after transplantation.

Donor-specific antibodies (DSA) were detected in five patients early after transplantation but disappeared or decreased significantly within the first 6 months after HTX. In one of these patients, histological findings revealed antibody-mediated rejection (1H) without increase of DSA in the first two biopsies of one patient. In addition to steroid therapy (500 mg methylprednisolone i.v. for 3 consecutive days), immunoadsorption treatment was started due to reduced biventricular function, which resolved after seven courses. All consecutive biopsies were negative.

During the first year, the incidence of ACR according to ISHLT criteria (≥2R) was 14.3% (*n* = 4), all occurring within the first month post transplantation. None showed hemodynamic compromise. All were treated successfully with i. v. steroid (500 mg methylprednisolone for 3 consecutive days). No patient showed recurrent rejection, nor rebound of ACR, after the end of ECP therapy. In one patient, immunosuppression was switched from tacrolimus to cyclosporine due to suspected tacrolimus-associated hyponatremia, 18 months post transplant. The patient consecutively developed ACR (ISHLT 2R) 3 months post conversion.

### Recurrence of Malignancy

After a median follow-up of 24.1 months (13.8–43.0), all patients are free of cancer without cancer recurrence, except the two patients with malignant cardiac tumor as indication for HTX: one patient died due to disease progression 13.3 months after transplant, and the other is in good clinical condition after post-transplant hepatic metastasectomy and chemotherapy 59.5 months after HTX.

## Discussion

In this hypothesis-generating study including 28 selected high-risk HTX patients, an ECP protocol first described by Barr et al. (14) and accompanied by a reduced-intensity immunosuppressive protocol was successfully applied. The safety and efficacy of this protocol in challenging HTX patients were confirmed.

Due to constant improvements in the results of HTX, the number of high-risk patients eligible for transplantation has increased significantly (17). Recent changes in allocation policies benefit patients in more unstable pre-transplant conditions partly bridged with temporary mechanical assist devices or ventricular assist device (VAD) complications (infection) (18–20). Moreover, patients with a history of cancer, even cardiac cancers, are considered potential candidates for transplantation in many centers (2, 21, 22). However, the preoperative condition of a patient has been shown to be directly associated with risk of severe infection and mortality (1, 12). Several scores have been established to predict post-transplant survival based on the preoperative condition (23–25). The Index for Mortality Prediction After Cardiac Transplantation (IMPACT) score has been validated with United Network for Organ Sharing (UNOS) data and includes pre-transplant risk factors like infection, short term mechanical assist devices, and others (23). Data of the Spanish National Heart Transplant Registry revealed an association between preoperative Interagency Registry for Mechanically Assisted Circulatory Support (INTERMACS) profiles and in-hospital mortality post-transplantation (1). Both reports have found infectious complications as one of the major causes of death post-transplantation (1, 17).

In our cohort, risk of early mortality was high, as 50% were already admitted to an intensive care unit before HTX and 25% were supported with temporary mechanical assist devices. This observation is supported by their high median IMPACT score of 8 (IQR 5.8–13).

Furthermore, patients with history of cancer might have a higher risk of developing malignancies after transplantation (2, 4). Overall immunosuppressive burden, time interval between pre-transplant cancer and transplantation, and cancer type seem to promote cancer development post-transplant (2, 4).

There is a general consensus that higher levels of immunosuppression are associated with a higher risk of infectious complications (26, 27). Moreover, critically ill patients seem to be immunocompromised (28). Therefore, it might be reasonable to aim for lower levels of immunosuppression after transplantation in patients at risk (5). However, strategies that avoid, delay, or minimize CNI use early after transplantation have shown higher rejection rates and the need for cytolytic antibody therapy, which bears a risk of infectious complications (5, 6, 29–31). On the other hand, immune monitoring of transplant patients has shown promising results, but never has reached routine clinical use (32).

ECP is an established therapy for the treatment of acute and chronic graft-versus-host disease after hematopoietic cell transplantation and rejection of solid organ transplantation and has been used for at least 25–30 years for these indications (33, 34). Nevertheless, the complete mode of action has not yet been elucidated. An increase in regulatory T cells and plasmacytoid dendritic cells has been observed during the use of ECP, which might have an immunomodulatory effect that leads to a more tolerogenic state of the immune system (35). Urbani et al. showed improved survival in high-risk liver transplant patients treated with ECP in combination with a CNI-sparing protocol, in comparison with a historical control group receiving standard triple immunosuppression (36). Although they partly failed in their main purpose of reducing CNI-induced toxicity, they did observe low infection rates and no deaths due to infection, compared with 16.5% in the control group. CNI was delayed by an average of 12.9 days. Acute rejection rates were numerically increased and rejection showed up earlier, which might have been due to the shorter duration of ECP therapy in combination with CNI delay. Only one prospective study has examined ECP early after HTX, comparing triple-drug immunosuppression with or without 6 months of ECP therapy (13). Barr et al. observed significantly lower rejection rates, similar overall infection, and lower CMV infection rates in the ECP group compared with the control group. No increase in rejection was detected after the end of ECP treatment. In both studies, ECP was tolerated very well and not associated with adverse events.

Based on the experience of those two studies, we developed our alternative protocol to test in three high-risk groups (infection, bridge to transplant *via* ECMO, history of cancer). We aimed to analyze the safety and efficacy of this protocol before starting a prospective randomized trial comparing this protocol with standard immunosuppression in control groups. We decided to combine the ECP protocol of Barr et al. with a reduced-intensity immunosuppressive protocol consisting of CNI delay (median of 3 days), lower target levels of tacrolimus (8–10 mg/ml instead of 12–15 ng/ml) and delayed steroid therapy at a lower dose (start: day 7 with 0.2 mg/kg instead of 1 mg/kg). MMF use was similar to that in our routine protocol. We applied no ATG induction therapy in all but the two of our first patients (100mg ATG once on POD1), assuming this would lower the risk of severe infections early after transplantation without risking higher rates of rejection. We trusted that a combination of tacrolimus/MMF would be more effective than cyclosporine/azathioprine, even at lower tacrolimus target levels and with delayed start of tacrolimus and oral steroid. Therefore, we defined as a secondary outcome an acceptable rate of acute rejections in the first year after transplantation as a rate 1.3–1.5-fold higher than the observed rejection rate with our conventional immunosuppressive protocol (15–20%) (37). This target rejection rate was similar to rejection rates in several other published studies over the last 10–15 years (20–25% rejection) (17, 38, 39, 45). Moreover, we assumed that lower-intensity immunosuppression without induction therapy with ATG might have a protective effect against cancer recurrence.

One-year survival in our high-risk patient cohort was slightly lower than in the overall patient cohort transplanted in the same time period (88.5% vs 93%). Nevertheless, risk-adjusted patient survival calculated using the IMPACT score was better than expected (88.5% vs 84.6%). Surprisingly, our patients with the highest predicted mortality (ECMO bridging to HTX) had 100% survival, compared with 71% expected survival. Patients with pre-transplant infections did worse than expected (75% vs 86% survival) but two patients died in the immediate postoperative period from fungal infection with strains resistant to empirically administered antifungal therapy. Both had developed grade III primary graft dysfunction (40). Whether the complicated postoperative course with primary graft dysfunction and ECMO additionally increased the risk of infection is an open question.

The incidence of severe infections in our cohort was 17.9% (*n* = 5), and they were lower respiratory tract infections necessitating prolonged ventilation or reintubation. Three of them were in the pre-transplant infection cohort. The lower overall rate of severe infections was surprising, as 66.7% of our patients had elevated risk due to infection and/or ECMO support pre-transplant. Nevertheless, our data are in accord with earlier reports showing that ECP after HTX is not associated with higher rates of infection despite earlier concerns about ECP leading to potential T-cell damage with subsequent reduced immune defense (14, 41, 42).

An unexpected finding was the low rate of ACR (14.3%) in the first year, all occurring in the first month. ACR episodes were without hemodynamic compromise. ACR was not associated with lower tacrolimus levels. Tacrolimus was delayed until a median of 3 days after transplantation, and the target range was reached at the end of the first week. Median achieved tacrolimus levels were in the upper target range over the first year, and this might have contributed to the low rejection rates. Nevertheless, maintenance steroids were started on day 7, at a lower dosage as recommended by guidelines, and were tapered until the end of the first year (34).

Most prospective randomized immunosuppressive trials in heart transplantation have reported an acute rejection rate of 15–25% during the first year (39, 43, 44). Based on previous reports, we assume that our ECP protocol had an impact on the low rejection rates (7, 14). Barr et al. showed a reduction from 82% to 61% of patients with at least one rejection episode when ECP was added to an immunosuppressive protocol consisting of cyclosporine and azathioprine (14). Similarly, we did not observe any rebound of acute rejection after the end of ECP therapy (14). Only one patient had a rejection episode during long-term follow-up, after switching from tacrolimus to cyclosporine for immunosuppression, on day 660. We can only speculate whether ECP induction treatment would allow even further decrease in overall immunosuppression early after transplantation. There is not enough evidence to proof that this protocol is safe in immunological high-risk patients.

Our eight transplant patients with a high neoplastic risk were heterogeneous: five had a prior history of cancer (three hematologic, one renal cancer, one with osteosarcoma and breast cancer), two had cardiac sarcoma at the time of transplantation, and one received a heart from a donor with lung cancer detected after procurement. Those with a history of cancer were cancer free for at least 5 years pre-transplant. In a retrospective analysis of 111 thoracic transplant patients from northern European centers, time from cancer detection to transplantation had an impact on cancer-free post-transplant outcomes and survival (21). Shorter time between cancer and transplant was associated with higher post-transplant cancer rates and worse outcome (21). Our patient cohort showed a similar pattern, with no post-transplant recurrence in all patients with ≥5 years after cancer detection, whereas both patients with sarcoma of the heart showed re-emergence of cancer within 1 year, leading to death in one of them. In a UNOS registry analysis, Yoosabai et al. reported a higher risk of post-transplant cancer and a median time of 3.2 years until cancer development in patients with pre-transplant cancer history (45). The low cancer rate we observed in this study might have been influenced by the short median follow-up of 20.7 months.

### Limitations

This is a hypothesis-generating study describing the outcome of a heterogeneous pilot group. Longer follow-up is needed to evaluate the incidence of cancer recurrence in patients with history of cancer. There is a strong need to compare our approach in a prospective randomized study with control groups for each indication.

### Conclusion

To our knowledge, this is the first description of the use of prophylactic ECP as an additional immunomodulatory therapy combined with reduced-intensity immunosuppressive maintenance therapy. There are no published data on a comparable protocol in HTX patients. In our heterogeneous pilot group of high-risk HTX patients, this innovative approach was safe, with low overall risk of rejection, and an effective strategy to address their high risk of infection or malignancy. Based on our data, future studies should be undertaken in a prospective randomized setting.

## Data Availability

The raw data supporting the conclusion of this article will be made available by the authors, without undue reservation.

## Abbreviations

AMR, antibody-mediated rejection; ACR, acute cellular rejection; CDC, complement-dependent cytotoxicity; CMP, cardiomyopathy; CNI, calcineurin inhibitor; CTCAE, common terminology criteria for adverse events; DSA, donor-specific antibody; ECMO, extracorporeal membrane oxygenation; ECP, extracorporeal photopheresis; HLA, human leucocyte antigen; HOCM, hypertrophic obstructive cardiomyopathy; HTX, heart transplantation; ICU, intensive care unit; INTERMACS, interagency registry for mechanically assisted circulatory support; IMPACT, index for mortality prediction after cardiac transplantation; IQR, interquartile range; ISHLT, international society of heart and lung transplantation; MMF, mycophenolate mofetil; MNC, mononuclear cells; MOF, multiorgan failure; PCR, polymerase chain reaction; POD, postoperative day; PRAs, panel-reactive antibodies; UNOS, united network for organ sharing; VAC, vacuum-assisted closer; VAD, ventricular assist device.
